# Glyphosate exposure, bone mineral density, osteoporosis, and fractures in the United States population

**DOI:** 10.1007/s40201-025-00969-7

**Published:** 2026-01-16

**Authors:** Naila Khalil, Latifa Hamdaoui, Alan Ducatman, Antti Koskela, Tarek Rebai, Miryoung Lee, Ramzi W. Nahhas

**Affiliations:** 1 Department of Population and Public Health Sciences, Boonshoft School of Medicine, Wright State University, 2555 University Blvd., Suite 210, Fairborn, OH 45324 USA; 2 Laboratory of induced and developmental diseases LR19ES12, Sfax Faculty of Medicine, University of Sfax, Sfax, 3029 Tunisia; 3 School of Public Health, West Virginia University, Morgantown, WV 26506 USA; 4 Research Unit of Translational Medicine, Faculty of Medicine, University of Oulu, Wellbeing Services County of North Ostrobothnia, Oulu, Finland; 5 Department of Epidemiology, Human Genetics and Environmental Sciences, The University of Texas Health Sciences Center at Houston, 780 Ringgold Road, Brownsville, TX 78520‑4979 USA; 6 Department of Psychiatry, Boonshoft School of Medicine, Wright State University, 2555 University Blvd., Suite 210, Fairborn, OH 45324 USA

**Keywords:** Bone mineral density, Osteoporosis, Sex differences, Fractures, Menopause, Children

## Abstract

**Purpose:**

Following reports of an adverse link between glyphosate and bone mineral density (BMD), this cross-sectional study extended the investigation between glyphosate exposure and BMD to include osteoporosis, and fractures, with a focus on the high risk, post-menopausal female subgroup.

**Methods:**

Cross-sectional data from 2,710 participants of the US National Health and Nutrition Examination Survey aged 8–59 years from 2013 to 2018 were used to assess associations between urinary glyphosate concentration (µg/L) and BMD (g/cm^2^) was measured using dual-energy X-ray absorptiometry (DXA), while osteoporosis and fractures were self-reported. Linear and logistic regression analyses were adjusted for age, race/ethnicity, BMI, smoking status, and income.

**Results:**

There was a negative association between glyphosate and BMD in the entire population (Whole Body BMD slope = -0.018; 95% CI = -0.027, -0.010; *p* < 0.001; Lumbar Spine BMD slope = -0.019; 95% CI = -0.030, -0.007; *p* = 0.002). In the high-risk, post-menopausal female subgroup, there was an inverse relationship (Whole Body BMD slope = -0.044; 95% CI = -0.076, -0.012; *p* = 0.009; Lumbar Spine BMD slope = -0.048; 95% CI = -0.101, 0.004, *p* = 0.069). Additionally, post-menopausal females with higher glyphosate had greater odds of self-reported osteoporosis (adjusted odds ratio (AOR) = 2.57; 95% CI = 1.24, 5.34; *p* = 0.011) and of self-reported fracture (AOR = 2.14; 95% CI = 1.28, 3.58, *p* = 0.004).

**Conclusions:**

Results are internally consistent and align with existing literature concerning toxicity of glyphosate on bone and extend it to include adverse impact on osteoporosis and fractures. Given the cross-sectional design and single point of sampling, longitudinal studies with repeated sampling are recommended to evaluate causation.

## Introduction

Glyphosate and glyphosate-based herbicides (GBH) are extensively applied herbicides internationally. They have been detected widely in ecosystems, domestic animals, wildlife, and humans [1–3]. Human exposure occurs primarily through air, food, and water intake [4], although dermal exposure is also possible [5]. Glyphosate is detectable in human serum, milk, and urine in both occupational cohorts and the general populations, with higher concentrations seen in males, children, and farmers [4–6]. Following human exposure, glyphosate accumulates principally in the kidneys, liver, colon [5], and small intestine and is eliminated in the feces (90%) and urine within 48 h. Although urine is not the major excretion pathway for glyphosate, the very short half-life has favored urine measures, and there is no reliable information concerning serum concentrations in the US population [7]. Thus, the United States [8] (US) National Health and Nutrition Examination Survey (NHANES) relies on urine measures for glyphosate assessment; [8].

A growing literature describes the adverse health impact of glyphosate on human population [9], including its adverse impact on the endocrine system [10], neurological outcomes [11], mortality [12], and liver pathology [13, 14], with inconclusive evidence regarding cancer [15]. Two longitudinal studies of human populations observed increasing temporal trends in detectable levels of glyphosate in urine [16]. 

Evidence regarding the impact of glyphosate on the skeletal health of humans is limited. Recent research detected a negative association between glyphosate exposure and total BMD in adults aged 20 to 59 years using NHANES 2013–2016 data [17]. Animal models report adverse effects of glyphosate on bone integrity, providing experimental plausibility [18].

The purpose of this cross-sectional study of NHANES 2013–2018 participants was to estimate associations between urinary glyphosate and BMD across a broader range of NHANES data and extend it to include highly relevant downstream outcomes of osteoporosis and fractures. While osteoporosis affects males and females across the age spectrum, post-menopausal females are at high risk for fragility fractures [19]. Post-menopausal estrogen deficits favor osteoclast synthesis and activity, while osteoblast activity decreases [20]. Therefore, we explored age and sex-specific associations between glyphosate and bone health. We hypothesized that a negative association of glyphosate with BMD will co-occur with an elevated risk of osteoporosis and fractures, and that all associations will differ by age and sex.

## Methods

### Study population and design

**Fig. 1:**
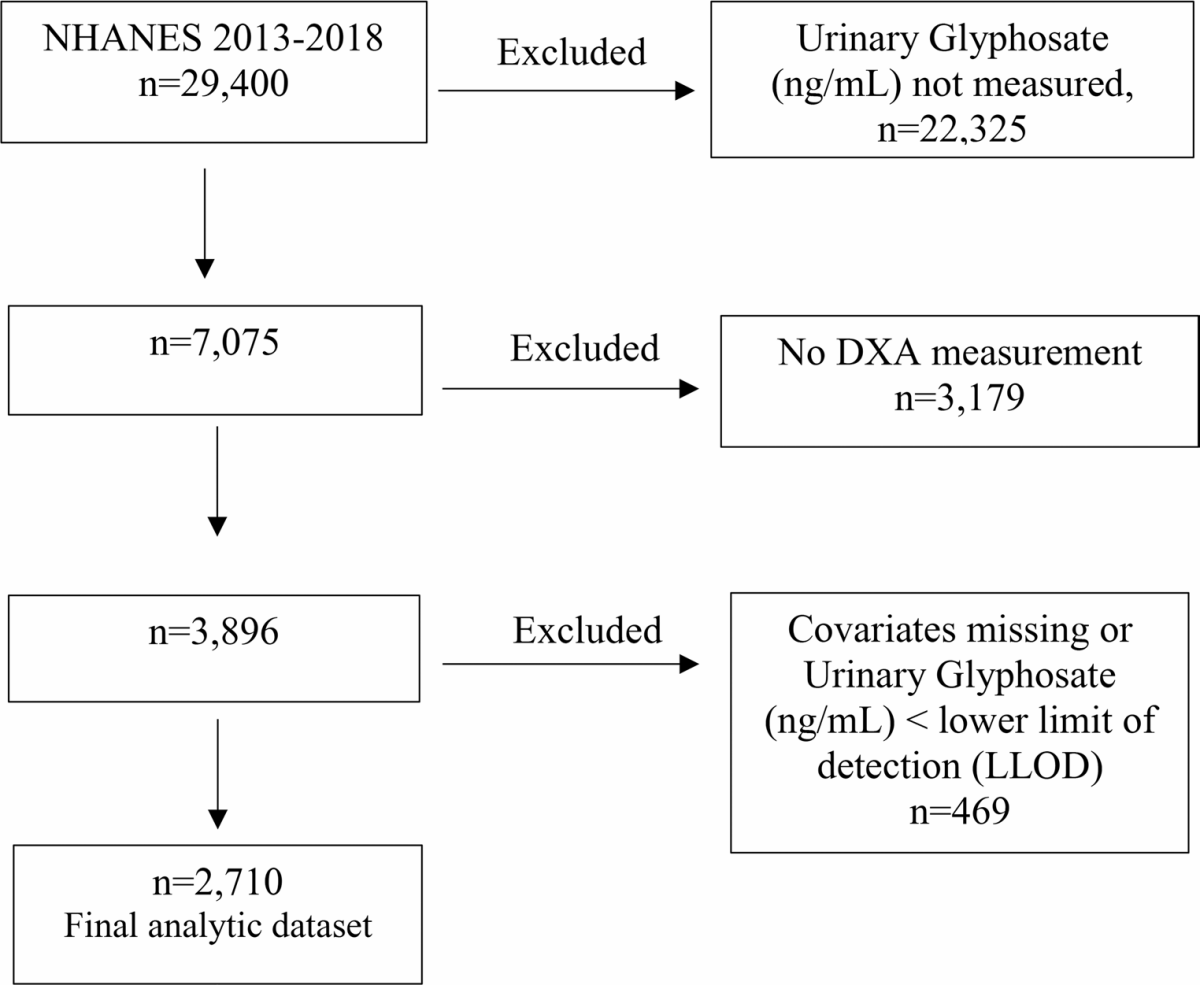
Flowchart showing selection of analytic dataset based on participants enrolled in the National Health and Nutrition Examination Surveys (NHAES) 2013–2018

NHANES 2013–2018 publicly available data were pooled over three two-year survey cycles and utilized for this study. Survey design details and methodology can be accessed on the NHANES website [21]. NHANES is an ongoing survey of the non-institutionalized US population that deploys a stratified, multistage probability sampling protocol to obtain data from a representative sample, after acquiring written informed consent from all participants. US National Center for Health Statistics (NCHS) Ethics Review Board (ERB) reviews and approves NHANES protocols to ensure protection of participants [21]. A flow chart is provided (Fig. 1.) showing selection of analytic dataset based on participants enrolled in the National Health and Nutrition Examination Surveys (NHAES) 2013–2018.

### Urinary glyphosate assessment

NHANES participants provided one spot urine sample (not strictly a first morning sample) and reported fasting status during a mobile examination center (MEC) visit [4]. Glyphosate was analyzed in stored spot urine samples in a random one-third representative subsample of participants aged 6 years and older from the three NHANES survey cycles [22]. A brief summary of methods follows.

Urine collected at the MEC was frozen onsite and shipped overnight on dry ice to the Centers for Disease Control’s (CDC) National Center for Environmental Health (NCEH) where they were stored at − 70 °C until analysis. About 200 µl of urine was used for this analysis which includes 2D-on-line ion chromatography coupled with tandem mass spectrometry (IC- MS/MS) and isotope dilution quantification [22]. For quality assurance, accuracy, and reliability, NHANES includes high- and low-concentration quality control materials and reagent blanks during each sample analysis [23].

### Bone mineral density assessment

Dual-energy X-ray absorptiometry (DXA) scans of the whole body (head, limbs, and torso including lumbar spine) were administered by trained and certified radiology technologists in the MEC to eligible survey participants aged 8 to 59 years. Whole-body scans were conducted on Hologic Discovery model A densitometers (Hologic, Inc., Bedford, Massachusetts) using software version Apex 3.2. Lumbar spine regions (from the whole-body scans) were analyzed separately as they provide information on osteoporosis and low bone mass for the sensitive population of participants ages 50 years and older [24]. A high level of quality control was maintained throughout the DXA data collection and scan analysis, including a rigorous phantom scanning schedule. To ensure the accuracy and consistency of the BMD results (g/cm^2^), the quality control center at the University of California, San Francisco (UCSF), Department of Radiology, used standard radiologic techniques and study-specific protocols developed for NHANES. Details of the DXA examination protocol are available on the NHANES website in the Body Composition Procedures Manual [24]. The number of participants who had whole body BMD DXA scan and lumbar spine DXA scans are different even though those are from the same scan due to variability in scans that met NHANES quality assurance standards. Valid data were available for 2,710 participants for lumbar spine bone mineral density, and 2440 participants for whole body bone mineral density.

### Self-Reported osteoporosis and fracture history assessment

Questionnaires were used to elicit self-reported osteoporosis and fracture history. These questions were asked only in the NHANES 2013-14 and 2017-18 survey cycles, and not in the NHANES 2015-16 cycle. Osteoporosis was coded as present if participants answered “yes” to the following question: “Has a doctor ever told you that you had osteoporosis, sometimes called thin or brittle bones?”. The validity of self-reported osteoporosis ranges from moderate to good among middle age and older adults, respectively [25]. Fractures were ascertained as “self-reported physician-diagnosed” if participants answered “yes” to any of the following questions: “Has a doctor ever told you that you had broken or fractured your hip/wrist/spine” or “Has a doctor ever told you that you had broken or fractured any other bone(s) after you were 20 years of age?”. Self-reported fractures are reasonably valid and provide accurate information on fractures in community-dwelling individuals with an 11% to 13% false positive rate [26, 27].

### Covariates

Based on literature, covariates were selected as a priori if they confound the relationship between exposure (glyphosate) and outcome (bone density, osteoporosis and fractures) [28]. Urinary glyphosate concentration differs by age, sex, race, and income [4] and bone density, osteoporosis and fractures risk are associated with increasing age, female sex, and white race [29]. Higher BMI is correlated with greater BMD, and fewer fractures [29]. Smokers have a higher risk of fractures and have a lower bone mass [29]. Literature also suggests that these covariates are correlated to glyphosate levels or, particularly, BMI play a mediating role in the association between glyphosate levels and chronic outcomes [30]. Dietary data was not included in the analysis since this information, captured in NHANES as self-reported 24-hour dietary recall, is considered controversial for the use of chronic disease adjustments [31].

Physical activity data was not included as physical activity varies widely across the broad age range (8 to 59 years) included in this study (children and adults), and physical activity levels (e.g., vigorous or leisure activity) were not uniformly collected across the included exam cycles. Further, as confounder, it is documented that physical activity is associated with improved skeletal health (increase bone mass, and lower risk of osteoporotic fractures) [32], however relationship between glyphosate and physical activity is not very clear; in one of the two studies currently addressing this topic physical activity and BMI interacted to impact urinary glyphosate levels [33].

Confounders in this analysis included age (years; continuous), race/ethnicity (non-Hispanic White, non-Hispanic Black, Mexican-American/Hispanic, non-Hispanic Asian, and “other” race including multi-racial), body mass index (BMI; kg/m^2^, continuous), annual household income (<$25,000, $25,000 to <$55,000, and $55,000+), and smoking status (based on serum cotinine) [34, 35]. If annual household income was missing, annual family income was used instead. NHANES obtains self-reported socio-demographic information via an interviewer-administered questionnaire. Serum cotinine levels < 1.0 ng/mL were categorized as non-smoker and 1.0 and above ng/mL as environmental tobacco smoke (ETS) or current smoker (as one group) [36].

NHANES 2013–2014, 2015–2016, and 2017–2018 have publicly available data on urinary creatinine concentrations which were analyzed using a commercially available enzymatic assay [22]. To account for urinary dilution, glyphosate, measured in micrograms per liter of urine (µg/L), was adjusted for creatinine as micrograms per gram of creatinine (µg/g creatinine) as published by Ospina et al. [4, 37], Menopause status was ascertained by questionnaire as self-reported cessation of regular menstruation over the past year. Females were categorized as postmenopausal if they answered “no” to a question “have you had regular periods in the past twelve months?” and specified the reason for not having regular periods was due to “menopause/hysterectomy”.

### Statistical Analysis

Pregnant females (positive urine pregnancy test and/or self-report) were ineligible for the DXA examination. Participants were excluded (for reasons other than pregnancy) from the DXA scan if they had an invalid scan, self-reported weight > 450 pounds, height over 6’5” (DXA table limitation), or self-reported history of radiographic contrast material (barium) use in past 7 days. In this analysis the lumbar spine bone mineral density from whole body DXA scan was used as a surrogate measure for posterior-anterior lumbar spine (L1-4) BMD.

According to the International Society for Clinical Densitometry 2019 Official Position for Pediatrics, the posterior-anterior lumbar spine and Whole Body less head, are the preferred skeletal sites for performing DXA measurements in children [38]. For whole body DXA scans in children, the contribution of head BMD to Whole Body BMD is disproportionately large, so the scan is reported ‘minus head’ Whole-Body BMD minus Head BMD, and we included whole body less head for children in the analysis.

Although diet and physical activity were not included due to concerns about overadjustment and measurement error, these factors may still act as potential confounders. Therefore, some degree of residual confounding cannot be excluded.

### Characteristics of the study population

Population characteristics, outcomes, and exposures are summarized using survey-weighted means and standard errors (SE) for continuous variables and sample frequencies and survey-weighted proportions for categorical variables. Descriptive statistics are presented overall, and by sex and age group. Unadjusted sex differences in participants’ characteristics were examined overall and within each age group using survey-weighted Rao-Scott chi-square tests for categorical variables and survey-weighted linear regression for continuous variables.

### Association between creatinine-adjusted glyphosate and BMD

Survey-weighted linear regression was used to estimate the association between creatinine-adjusted glyphosate with total/whole body BMD and lumbar spine BMD. Due to a highly skewed distribution, a natural log-transformation was applied to creatinine-adjusted glyphosate (log-creatinine-adjusted glyphosate) prior to analysis. Additionally, analyses were repeated by grouping participants into creatinine-adjusted glyphosate quartiles (Q1: referent, lowest; Q4: highest). creatinine-adjusted glyphosate quartile cutoffs were computed for each analysis individually so they would be based on the exact same sample used in the analysis of continuous log-creatinine-adjusted glyphosate. Multivariable linear regression models included whole body bone mineral density, and lumbar spine bone mineral density as dependent variables, and log-creatinine-adjusted glyphosate as continuous predictor or in quartiles.

Analyses were done overall (males and females pooled together) and by sex (male, female) for all participants (age 8–59 years), adults (age 18–59 years), and children and adolescents (age 8–17 years). Additionally, analyses were conducted among adult females overall (adjusted for self-reported menopausal status) and stratified by self-reported menopausal status, as menopause is a major risk factor for low BMD and osteoporosis [19].

Survey-weighted logistic regression was used to estimate the association of log-creatinine-adjusted glyphosate (and creatinine-adjusted glyphosate quartiles) with self-reported osteoporosis among post-menopausal females, and with self-reported fracture among all, pre-menopausal, and post-menopausal adult females. Analyses of osteoporosis among all and pre-menopausal adult females were omitted due to a very low prevalence of osteoporosis among pre-menopausal females. Additionally, there were no post-menopausal females in the second creatinine-adjusted glyphosate quartile who reported having an osteoporosis diagnosis, resulting in quasi-separation in the logistic regression, so the first and second quartiles were combined for that specific analysis.

All analyses were adjusted for age, race/ethnicity, BMI, smoking status, and income. Due to violations of the linearity assumption for age and BMI, we additionally adjusted for age [2] and BMI [2]. Analyses pooled over sexes were additionally adjusted for gender. Analyses of fractures among all adult females were adjusted for menopausal status. Due to sparsity among those with the outcome, the race/ethnicity variable was collapsed into white vs. all others for the analyses of osteoporosis and fractures.

SAS survey procedures were used to account for the complex NHANES survey design (SAS Institute, Inc., version 9.4). Specifically, SURVEYMEANS and SURVEYFREQ were used for descriptive statistics and SURVEYREG and SURVEYLOGISTIC were used for linear and logistic regressions, respectively. R 4.3.2 [39], and the survey library [40], were used to check regression assumptions (normality, constant variance, linearity) and conduct diagnostics (collinearity based on variance inflation factors, outliers based on a Bonferroni outlier test, and influence based on Difference in Betas [DFBetas]). All tests were two-sided and at the 5% level of significance. (Tables 1 and 2)

**Table 1:** Sample frequencies (N) and estimated descriptive statistics for the target population enrolled in the National health and nutrition examination surveys 2013–2018, age 8 to 59 years, overall and by sex

	Overall *N* = 2710	Female *N* = 1265 (47%)	Male *N* = 1445 (53%)	Female vs. Male
Characteristic	Mean ± SE or N (%)	Mean ± SE or N (%)	Mean ± SE or N (%)	p
Urinary glyphosate (µg/L)	0.603 (0.026)	0.618 (0.035)	0.589 (0.025)	0.349
Urinary creatinine-adjusted glyphosate (µg/g)	0.524 (0.016)	0.610 (0.029)	0.449 (0.016)	< 0.001
Lumbar spine BMD (g/cm^2^)	0.996 (0.006)	1.002 (0.008)	0.990 (0.006)	0.170
Whole BodyBMD (g/cm^2^) ^a^	1.043 (0.006)	1.009 (0.007)	1.073 (0.007)	< 0.001
Age (years)	32.8 (0.4)	33.3 (0.6)	32.4 (0.5)	0.281
BMI (kg/m^2^)	27.7 (0.2)	27.8 (0.3)	27.6 (0.2)	0.529
Smoking status				
Non-smoker	1987 (73.2)	998 (78.2)	989 (68.9)	< 0.001
ETS/Current smoker	723 (26.8)	267 (21.8)	456 (31.1)	
Race/ethnicity				
NH White	952 (60.0)	429 (60.1)	523 (59.9)	0.455
NH Black	590 (12.6)	289 (13.2)	301 (12.2)	
Hispanic	736 (17.9)	348 (16.7)	388 (18.9)	
NH Asian	277 (4.7)	126 (4.7)	151 (4.8)	
Other	155 (4.8)	73 (5.3)	82 (4.3)	
Annual Income				
<$25,000	952 (60.0)	319 (17.7)	353 (17.3)	0.522
$25,000 to <$55,000	590 (12.6)	431 (29.6)	461 (27.4)	
$55,000+	736 (17.9)	515 (52.7)	631 (55.2)	

All reported statistics are survey weighted including mean, standard error (SE), proportion (%), p-value (p). P-values are based on survey-weighted Rao-Scott chi-square tests for categorical variables and survey-weighted linear regression for continuous variables

^a^ In children and adolescents, Whole/total Body BMD excludes head BMD (Whole Body Less Head BMD, g/cm2]). The sample sizes for Whole Body BMD are 2440 (Overall), 1144 (Female), and 1296 (Male)

**Table 2:** Sample frequencies (N) and estimated descriptive statistics for the target population enrolled in the National health and nutrition examination surveys 2013–2018, by age group and sex

	Adults (18–59 years) *N* = 1792			Children and Adolescents (8–17 years) *N* = 918		
	Female *N* = 846 (46%)	Male *N* = 946 (54%)	Female vs. Male	Female *N* = 419 (48%)	Male *N* = 499 (52%)	Female vs. Male
**Characteristic**	Mean ± SE or N (%)	Mean ± SE or N (%)	p	Mean ± SE or N (%)	Mean ± SE or N (%)	p
Urinary glyphosate (µg/L)	0.604 (0.043)	0.552 (0.029)	0.168	0.665 (0.033)	0.726 (0.036)	0.130
Urinary creatinine-adj. glyphosate (µg/g)	0.611 (0.035)	0.407 (0.016)	< 0.001	0.605 (0.033)	0.601 (0.041)	0.930
Lumbar spine BMD (g/cm^2^)	1.041 (0.007)	1.034 (0.007)	0.402	0.869 (0.015)	0.833 (0.010)	0.072
Whole BodyBMD (g/cm^2^) ^a^	1.075 (0.005)	1.144 (0.007)	< 0.001	0.806 (0.010)	0.836 (0.009)	0.012
Age (years)	39.4 (0.6)	37.9 (0.6)	0.071	12.4 (0.2)	12.7 (0.2)	0.432
BMI (kg/m^2^)	29.4 (0.4)	29.0 (0.2)	0.366	22.1 (0.4)	22.2 (0.4)	0.828
Smoking status						
Non-smoker	629 (75.0)	569 (64.6)	< 0.001	369 (89.1)	420 (84.3)	0.173
ETS/Current smoker	217 (25.0)	377 (35.4)		50 (10.9)	79 (15.7)	
Race/ethnicity						
NH White	313 (61.6)	365 (61.7)	0.398	116 (54.9)	158 (53.2)	0.931
NH Black	176 (12.5)	183 (11.0)		113 (15.5)	118 (16.5)	
Hispanic	215 (15.7)	240 (18.4)		133 (20.2)	148 (20.8)	
NH Asian	96 (5.1)	109 (4.9)		30 (3.5)	42 (4.2)	
Other	46 (5.1)	49 (4.0)		27 (6.0)	33 (5.2)	
Annual Income						
<$25,000	212 (17.6)	237 (17.7)	0.325	107 (18.1)	116 (16.2)	0.762
$25,000 to <$55,000	281 (29.7)	288 (26.3)		150 (28.9)	173 (31.3)	
$55,000+	353 (52.6)	421 (56.0)		162 (53.0)	210 (52.5)	

All reported statistics are survey weighted including mean, standard error (SE), proportion (%), p-value (p). P-values are based on survey-weighted Rao-Scott chi-square tests for categorical variables and survey-weighted linear regression for continuous variables

^a^ In children and adolescents, Whole Body BMD excludes head BMD (Whole Body Less Head BMD, g/cm2]). The sample sizes for Whole/total Body BMD are 1574 (Adults), 751 (Adults, Female), 823 (Adults, Male), 866 (Children and Adolescents), 393 (Children and Adolescents, Female), and 473 (Children and Adolescents, Male)

### Sample size

The present study used data from 2,710 NHANES 2013–2018 participants, age 8**–**59 years, who had complete information available for whole body bone mineral density and/or lumbar spine bone mineral density, urinary glyphosate, and covariates (listed below), and 284/285 female participants, age 40–59 who had complete information for self-reported osteoporosis/fractures, urinary glyphosate, and covariates (Fig. 1). Overall, the 2,710 participants represent 88% of the 3,896 participants for whom both urinary glyphosate and DXA were measured; 12% had a missing value for outcome, exposure, or a confounder, or had exposure below the lower limit of detection (Fig. 1). Cases with missing values were omitted and complete case analyses were conducted. Sample sizes for specific analyses are shown in Tables 3, 4, 5, 6 and 7 and vary due to the possibility of having a valid lumbar spine bone mineral density measurement but an invalid whole-body bone mineral density measurement, self-reported osteoporosis and fractures not being available for 2015–2016, and stratification by age group, sex, or menopausal status.

**Table 3:** Survey-weighted linear regression analysis of bone mineral density on log urinary creatinine-adjusted glyphosate (log µg/g) (Exposure) in participants enrolled in the National health and nutrition examination surveys 2013–2018, age 8 to 59 years, overall and by sex

			All Participants, *N* = 2440		Females, *N* = 1144		Males, *N* = 1296	
Outcome	Exposure	Quartile	β (95% CI)	p	β (95% CI)	p	β (95% CI)	p
Whole body BMD (g/cm^2^)^a^	Linear		−0.018 (−0.027, −0.010)	< 0.001	−0.018 (−0.028, −0.008)	< 0.001	−0.017 (−0.029, −0.005)	0.005
	Quartile mean differences	Q1	Reference	0.009 ^b^	Reference	0.005 ^b^	Reference	0.084^b^
		Q2	−0.010 (−0.030, 0.010)	0.317	0.003 (−0.022, 0.028)	0.825	−0.010 (−0.035, 0.016)	0.454
		Q3	−0.019 (−0.035, −0.003)	0.018	−0.007 (−0.028, 0.014)	0.521	−0.017 (−0.044, 0.011)	0.229
		Q4	−0.033 (−0.053, −0.014)	0.001	−0.031 (−0.050, −0.012)	0.002	−0.031 (−0.056, −0.007)	0.014
			All Participants, *N* = 2710		Females, *N* = 1265		Males, *N* = 1445	
Lumbar spine BMD (g/cm^2^)	Linear		−0.019 (−0.030, −0.007)	0.002	−0.020 (−0.040, 0.000)	0.047	−0.018 (−0.032, −0.004)	0.012
	Quartile mean differences	Q1	Reference	0.013 ^b^	Reference	0.009^b^	Reference	0.174^b^
		Q2	−0.007 (−0.035, 0.020)	0.597	0.027 (−0.014, 0.069)	0.192	−0.016 (−0.049, 0.017)	0.340
		Q3	−0.012 (−0.034, 0.010)	0.284	0.007 (−0.029, 0.043)	0.683	−0.022 (−0.054, 0.011)	0.190
		Q4	−0.038 (−0.062, −0.013)	0.003	−0.035 (−0.068, −0.002)	0.037	−0.034 (−0.065, −0.004)	0.028

All models adjusted for age, age square [2], race/ethnicity, BMI, BMI square [2], smoking status, and income; models for “All Participants” additionally adjusted for gender)

^a^ In children and adolescents, Whole Body BMD excludes head BMD (Whole Body Less Head BMD, g/cm2])

^b^ The p-value next to “Reference” is for the 3 degrees of freedom test of the null hypothesis that the quartile means are all equal

**Table 4:** Survey-weighted linear regression analysis of bone mineral density on log urinary creatinine-adjusted glyphosate (log µg/g) (Exposure) in participants enrolled in the National health and nutrition examination surveys 2013–2018, for adults age 18 to 59 years, overall and by sex

			Adults, *N* = 1574		Females, *N* = 751		Males, *N* = 823	
Outcome	Exposure	Quartile	β (95% CI)	p	β (95% CI)	p	β (95% CI)	p
Whole body BMD (g/cm^2^)	Linear		−0.012 (−0.021, −0.003)	0.011	−0.014 (−0.025, −0.004)	0.009	−0.009 (−0.025, 0.006)	0.218
	Quartile mean differences	Q1	Reference	0.259^a^	Reference	0.034^a^	Reference	0.744^a^
		Q2	−0.006 (−0.026, 0.014)	0.550	0.005 (−0.021, 0.031)	0.701	−0.003 (−0.028, 0.022)	0.831
		Q3	−0.006 (−0.024, 0.013)	0.539	0.000 (−0.024, 0.024)	1.000	−0.012 (−0.044, 0.020)	0.448
		Q4	−0.018 (−0.036, 0.001)	0.064	−0.023 (−0.043, −0.004)	0.020	−0.015 (−0.044, 0.013)	0.285
			Adults, *N* = 1792		Females, *N* = 846		Males, *N* = 946	
Lumbar spine BMD (g/cm^2^)	Linear		−0.009 (−0.022, 0.004)	0.151	−0.013 (−0.035, 0.009)	0.237	−0.007 (−0.025, 0.011)	0.433
	Quartile mean differences	Q1	Reference	0.393^a^	Reference	0.039^a^	Reference	0.656^a^
		Q2	−0.004 (−0.031, 0.022)	0.752	0.036 (−0.009, 0.082)	0.116	−0.022 (−0.060, 0.015)	0.237
		Q3	−0.001 (−0.025, 0.024)	0.967	0.021 (−0.016, 0.057)	0.261	−0.018 (−0.056, 0.020)	0.351
		Q4	−0.019 (−0.044, 0.006)	0.135	−0.020 (−0.058, 0.017)	0.277	−0.017 (−0.053, 0.019)	0.346

All models adjusted for age, age square [2], race/ethnicity, BMI, BMI square [2], smoking status, and income; models for “Adults” additionally adjusted for gender)

^a^ The p-value next to “Reference” is for the 3 degrees of freedom test of the null hypothesis that the quartile means are all equal

**Table 5:** Survey-weighted linear regression analysis of bone mineral density on log urinary creatinine-adjusted glyphosate (log µg/g) (Exposure) in participants enrolled in the National health and nutrition examination surveys 2013–2018, for children and adolescents age 8 to 17 years, overall and by sex

			Children & Adolescents, *N* = 866		Females, *N* = 393		Males, *N* = 473	
Outcome	Exposure	Quartile	β (95% CI)	p	β (95% CI)	p	β (95% CI)	p
Whole body BMD (g/cm^2^)^b^	Linear		−0.007 (−0.016, 0.003)	0.172	0.002 (−0.008, 0.012)	0.713	−0.012 (−0.025, 0.001)	0.080
	Quartile mean differences	Q1	Reference	0.801 ^b^	Reference	0.833 ^b^	Reference	0.701 ^b^
		Q2	−0.002 (−0.020, 0.016)	0.852	0.010 (−0.013, 0.033)	0.385	−0.016 (−0.043, 0.012)	0.260
		Q3	0.000 (−0.019, 0.019)	0.991	0.011 (−0.016, 0.037)	0.431	−0.010 (−0.047, 0.026)	0.576
		Q4	−0.008 (−0.029, 0.012)	0.429	0.005 (−0.016, 0.027)	0.632	−0.014 (−0.046, 0.017)	0.370
			Children & Adolescents, *N* = 918		Females, *N* = 419		Males, *N* = 499	
Lumbar spine BMD (g/cm^2^)	Linear		−0.011 (−0.023, 0.001)	0.069	−0.008 (−0.028, 0.012)	0.409	−0.011 (−0.026, 0.004)	0.147
	Quartile mean differences	Q1	Reference	0.448 ^b^	Reference	0.287 ^b^	Reference	0.103 ^b^
		Q2	−0.014 (−0.044, 0.016)	0.357	0.019 (−0.023, 0.061)	0.370	−0.033 (−0.063, −0.002)	0.037
		Q3	−0.007 (−0.032, 0.019)	0.603	0.017 (−0.026, 0.060)	0.421	−0.029 (−0.066, 0.008)	0.117
		Q4	−0.021 (−0.049, 0.007)	0.141	−0.011 (−0.052, 0.029)	0.578	−0.019 (−0.055, 0.017)	0.292

All models adjusted for age, age square [2], race/ethnicity, BMI, BMI square [2], smoking status, and income; models for “Children & Adolescents” additionally adjusted for gender)

^a^ In children and adolescents, Whole Body BMD excludes head BMD (Whole Body Less Head BMD, g/cm2])

^b^ The p-value next to “Reference” is for the 3 degrees of freedom test of the null hypothesis that the quartile means are all equal

**Table 6:** Survey-weighted linear regression analysis of bone mineral density on log urinary creatinine-adjusted glyphosate (log µg/g) (Exposure) in participants enrolled in the National health and nutrition examination surveys 2013–2018, for adult females age 18 to 59 years, overall and by menopausal status

			Adult Females, *N* = 751		Pre-Menopausal, *N* = 570		Post-Menopausal, *N* = 181	
Outcome	Exposure	Quartile	β (95% CI)	p	β (95% CI)	p	β (95% CI)	p
Whole body BMD (g/cm^2^)	Linear		−0.014 (−0.025, −0.004)	0.008	−0.002 (−0.010, 0.006)	0.583	−0.044 (−0.076, −0.012)	0.009
	Quartile mean differences	Q1	Reference	0.022 ^a^	Reference	0.984 ^a^	Reference	0.002 ^a^
		Q2	0.006 (−0.020, 0.032)	0.661	0.001 (−0.025, 0.026)	0.952	−0.051 (−0.120, 0.017)	0.138
		Q3	0.000 (−0.025, 0.026)	0.976	0.001 (−0.023, 0.026)	0.910	−0.039 (−0.093, 0.015)	0.156
		Q4	−0.023 (−0.041, −0.004)	0.017	0.003 (−0.016, 0.022)	0.750	−0.118 (−0.187, −0.049)	0.001
			Adult Females, *N* = 846		Pre-Menopausal, *N* = 636		Post-Menopausal, *N* = 210	
Lumbar spine BMD (g/cm^2^)	Linear		−0.012 (−0.035, 0.010)	0.282	0.003 (−0.018, 0.024)	0.764	−0.048 (−0.101, 0.004)	0.069
	Quartile mean differences	Q1	Reference	0.032 ^a^	Reference	0.123 ^a^	Reference	0.187 ^a^
		Q2	0.037 (−0.008, 0.082)	0.107	0.010 (−0.024, 0.044)	0.563	−0.089 (−0.207, 0.029)	0.137
		Q3	0.022 (−0.016, 0.061)	0.249	0.034 (−0.001, 0.068)	0.057	−0.082 (−0.198, 0.033)	0.157
		Q4	−0.018 (−0.056, 0.020)	0.338	−0.002 (−0.042, 0.038)	0.916	−0.138 (−0.273, −0.003)	0.045

All models adjusted for age, age square [2], race/ethnicity, BMI, BMI square [2], smoking status, and income; models for “Adult Females” additionally adjusted for menopausal status)

^a^ The p-value next to “Reference” is for the 3 degrees of freedom test of the null hypothesis that the quartile means are all equal

**Table 7:** Survey-weighted logistic regression analysis of self-reported osteoporosis and fractures on log urinary creatinine-adjusted glyphosate (log µg/g) (Exposure) in participants enrolled in the National health and nutrition examination surveys 2013–2018, for adult females age 40 to 59 years, overall and by menopausal status

			Adult Females, *N* = 284 (Osteoporosis prevalence = 10.2%) ^a^		Pre-Menopausal, *N* = 127 (Osteoporosis prevalence = 4.3%) ^a^		Post-Menopausal, *N* = 157 (Osteoporosis prevalence = 15.0%) ^a^	
Outcome	Exposure	Quartile	Adjusted Odds Ratio (95% CI)	p^b^	Adjusted Odds Ratio (95% CI)	p^b^	Adjusted Odds Ratio (95% CI)	p^b^
Self-reported osteoporosis	Linear		2.190 (0.974, 4.921)	0.058	2.164 (0.246, 19.07)	0.487	2.574 (1.241, 5.338)	0.011
	Quartile comparisons	Q1 & Q2^c^		^d^		^d^	Reference	0.005
		Q3					8.247 (1.792, 37.96)	0.095
		Q4					9.104 (2.302, 36.00)	0.028
			Adult Females, *N* = 285 (fracture prevalence = 37.9%) ^b^		Pre-Menopausal, *N* = 127 (fracture prevalence = 29.5%) ^b^		Post-Menopausal, *N* = 158 (fracture prevalence = 44.5%)^b^	
Self-reported fracture	Linear		1.755 (1.165, 2.644)	0.007	1.155 (0.408, 3.268)	0.786	2.140 (1.281, 3.575)	0.004
	Quartile comparisons	Q1	Reference	0.136	Reference	0.934	Reference	0.019
		Q2	0.771 (0.254, 2.341)	0.187	1.430 (0.378, 5.413)	0.781	0.746 (0.180, 3.097)	0.083
		Q3	1.042 (0.437, 2.484)	0.570	1.147 (0.234, 5.634)	0.852	1.525 (0.395, 5.886)	0.930
		Q4	2.878 (0.994, 8.331)	0.025	1.531 (0.255, 9.184)	0.751	5.453 (1.475, 20.16)	0.003

All models adjusted for age, age square [2], BMI, BMI square [2], race/ethnicity (white vs. all others), smoking status, and income; models for “Adult Females” were additionally adjusted for menopausal status

^a^ Survey-weighted estimate of the population prevalence

^b^ The p-value next to “Reference” for self-reported fracture is for the 3 degree of freedom tests of the null hypothesis that the quartile means are all equal

^c^ There were no post-menopausal females in the second exposure quartile who reported having an osteoporosis diagnosis, resulting in quasi-separation, so Q1 and Q2 were combined

^d^ Only one pre-menopausal female in each exposure quartile reported having an osteoporosis diagnosis. Due to this level of sparsity, the pre-menopausal and overall quartile analyses were not done

## Results

### Characteristics of the study population

Descriptive characteristics in the BMD sample, overall (*n* = 2,710) and by sex, are presented in Table 1. Overall, the weighted mean (weighted standard error, SE) of urinary creatinine-adjusted glyphosate was 0.524 (0.016). While there was no difference in unadjusted glyphosate level (µg/L) between males and females (*p* = 0.349), the mean ± SE of creatinine-adjusted glyphosate was significantly greater in females (0.610 ± 0.029 µg/g) than in males (0.449 ± 0.016 µg/g) (*p* < 0.001).

The weighted mean ± weighted SE whole body bone mineral density in the overall sample was 1.043 ± 0.006 g/cm^2^ and whole-body bone mineral density in males (1.073 g/cm^2^) was significantly higher than in females (1.009 g/cm^2^) (*p* < 0.001). Weighted mean ± weighted SE regional lumbar spine bone mineral density in the overall sample was 0.996 ± 0.006 g/cm^2^ and the mean did not differ significantly between sexes (*p* = 0.170). There was no significant difference in age, BMI, race/ethnicity, or annual income levels between males and females. However, current smoking prevalence, based on serum cotinine, was significantly greater in males (31.1%) than in females (21.8%) (*p* < 0.001).

Characteristics by age group and sex are presented in Table 2. The sex difference in urinary creatinine-adjusted glyphosate seen when all ages were pooled, was present only in adults, and not in children/adolescents. Weighted mean whole-body bone mineral density differed between sexes in children and in adults, with whole body bone mineral density significantly greater in males than in females.

### Association between creatinine-adjusted glyphosate and BMD

Tables 3, 4, 5 and 6 display the survey-weighted adjusted linear regression results for all participants (Table 3), adults (Table 4), children and adolescents (Table 5), and adult females (Table 6). When examining all ages pooled together (Table 3), log-creatinine-adjusted glyphosate was significantly negatively associated with each of whole-body bone mineral density and lumbar spine bone mineral density, overall, and for males and females separately. Results from the creatinine-adjusted glyphosate quartile analysis are consistent with these results, showing, in general, lower mean BMD in higher glyphosate exposure quartiles.

When examining only adults (Table 4), log-creatinine-adjusted glyphosate remained negatively associated with both whole-body bone mineral density and lumbar spine bone mineral density. However, the magnitude of the association was weaker in adults compared to all ages pooled together (Table 3). Among adults, the reduced subgroup sample size and the weaker associations manifested as this relationship only being statistically significant for whole body bone mineral density overall (slope = −0.012; 95% CI = −0.021, −0.003; *p* = 0.011) and in females (slope = −0.014; 95% CI = −0.025, −0.004; *p* = 0.009). The creatinine-adjusted glyphosate quartile analysis indicates that an adverse effect of the exposure on BMD overall and in females is only meaningfully large among those in the highest quartile of exposure. Among females, those in the highest quartile of creatinine-adjusted glyphosate exposure have significantly lower mean whole-body bone mineral density than those in the lowest quartile (difference in means = −0.023; 95% CI = −0.043, −0.004; *p* = 0.020).

When examining children and adolescents (Table 5), log-creatinine-adjusted glyphosate was negatively associated with each of whole-body bone mineral density and lumbar spine bone mineral density among males, but log-creatinine-adjusted glyphosate was not significantly associated with either of BMD measures. The creatinine-adjusted glyphosate quartiles results indicated some non-linearity in the exposure-outcome association, however compared to the lowest exposure quartile, those in the highest quartile generally had a smaller mean BMD regardless of sex.

**Fig. 2:**
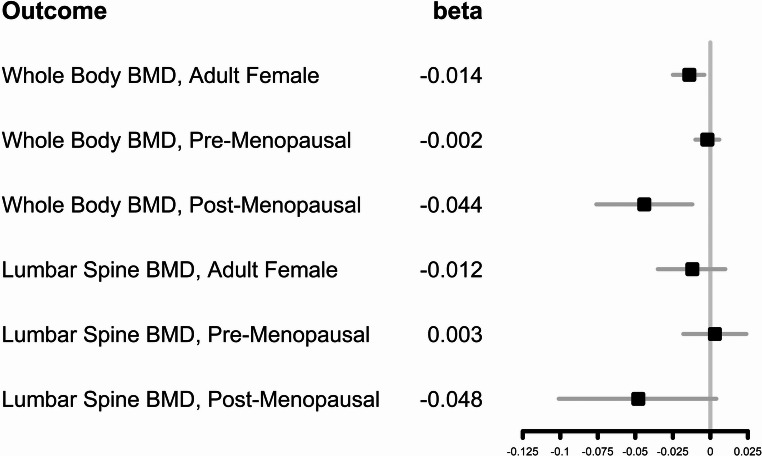
Forest plot of adjusted regression coefficients and their 95% confidence intervals (linear exposure models)

The adverse association of urine glyphosate to BMD among adult females was only meaningfully large among post-menopausal females (Table 6) and not significant among pre-menopausal females. A statistically significant larger magnitude association between log-creatinine-adjusted glyphosate and whole-body bone mineral density among post-menopausal females (whole body bone mineral density slope = −0.044; 95% ci = −0.076, −0.012; *p* = 0.009 was noted (lumbar spine bone mineral density slope = −0.048; 95% CI = −0.101, 0.004, *p* = 0.069). The adjusted regression coefficients for the linear exposure models and their 95% confidence intervals are illustrated in Fig. 2.

### Association between creatinine-adjusted glyphosate and osteoporosis and fractures

**Fig. 3:**
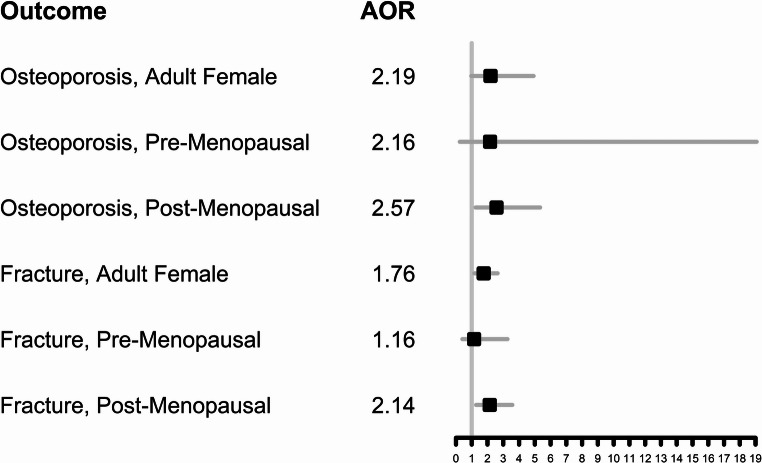
Forest plot of adjusted odds ratios and their 95% confidence intervals (linear exposure models)

Table 7 displays the survey-weighted adjusted logistic regression results for self-reported osteoporosis and fractures among adult females. As with the analysis of BMD (Table 6), there is a strong adverse association between the urine glyphosate concentration and both osteoporosis and fractures in post-menopausal females. Post-menopausal females who have one-unit higher log-creatinine-adjusted glyphosate had 2.57 times odds of osteoporosis (adjusted odds ratio (AOR) = 2.57; 95% CI = 1.24, 5.34; *p* = 0.011) and fracture (AOR = 2.14; 95% CI = 1.28, 3.58, *p* = 0.004). The creatinine-adjusted glyphosate quartile analysis was generally consistent with these results, with the largest effect seen in the highest creatinine-adjusted glyphosate quartile, especially for fractures. Among pre-menopausal females, the association was meaningfully large only for osteoporosis; however, due to the small sample size, this association was not statistically significant (AOR = 2.16; 95% CI = 0.25, 19.1, *p* = 0.487). The adjusted odds ratios for the linear exposure models and their 95% confidence intervals are illustrated in Fig. 3.

### Outliers and influential observations

The models each had from 0 to 7 outliers; however, given the sample sizes these do not influence the regression assumptions. Nevertheless, a few outliers were influential. Specifically, (1) for lumbar spine, females (Table 3), removal of two influential observations (DFBeta > 0.4 for the exposure) resulted in the regression coefficient being attenuated and no longer being statistically significant; (2) for self-reported osteoporosis, post-menopausal (Table 7), removal of seven influential observations resulted in the adjusted odds ratio being attenuated from 2.57 to 1.88 and no longer being statistically significant; and (3) for self-reported fracture, post-menopausal (Table 7), removal of two influential observations resulted in the adjusted odds ratio increasing from 2.14 to 3.30. In summary, the main findings of the paper regarding the association between glyphosate and BMD were not impacted by outliers or influential observations.

## Discussion

In this representative sample of the U.S. population, increasing urinary glyphosate concentration was adversely associated with adult BMD. These associations included whole body bone mineral density and lumbar spine bone mineral density pooled over sexes, as well in males and females separately. A similar association was evident in creatinine-adjusted glyphosate quartile analysis, in general showing lower mean BMD with higher exposure quartiles. The significant negative association seen in all females between log-creatinine-adjusted glyphosate and BMD was also present among post-menopausal females in stratified analysis. Similar trends were seen in children, but the associations were not statistically significant. In addition, the association between osteoporosis and urinary creatinine-adjusted glyphosate concentration was meaningfully large in both pre- and post-menopausal women, the effect was of larger magnitude (and statistically significant) in post-menopausal women. For fractures, however, the effect was only meaningfully large and statistically significant in post-menopausal women who are at high risk for fragility fractures.

An incidental finding of our analyses is the markedly greater impact of creatinine-adjustment on urine measures of glyphosate (creatinine-adjusted glyphosate) in males compared to females. Urinary creatinine tracks with muscle mass and is lower in women [41], so that is the likely explanation for the greater impact of the adjustment in men.

Wang et al. (2024) previously reported an adverse relationship between urinary glyphosate and whole-body bone mineral density in U.S. adult males and females using NHANES data from 2013 to 2016 [28]. We observed the same results as Wang et al., even though there were differences among these two studies regarding sample size, included age range, adjustment for urinary creatinine levels, and covariates. Our study reports for the first-time parallel associations with self-reported osteoporosis and fractures, especially among the high-risk population of post-menopausal females.

Experimental evidence supports consideration of these associations with imaging biomarkers of bone density, osteoporosis, and fractures as potentially causal. Glyphosate complexes with biogenic hydroxyapatite [42]. The total burden of glyphosate seven days after administration (approximately 1% of the administered dose) was principally absorbed in the bone tissue [43]. Existing experimental evidence reveals that glyphosate adversely impacts bone structure in female Wistar rats, as shown by alterations in biomarkers of bone metabolism such as alkaline phosphatase, as well as histologic findings of bone rarefaction, discontinuity of bone trabeculae, and a significant decrease in the number of intertrabecular links suggestive of osteoporosis [18]. In addition, computational modeling reveals that glyphosate binds strongly to bone proteins (osteocalcin) and may impair bone metabolism by affecting osteocalcin [44].

Bone formation and resorption are regulated by the interaction of estrogens, testosterone, thyroid and parathyroid hormone, vitamin D, and glucocorticoids, with close involvement of kidneys, intestines, liver, and several other metabolic pathways [45]. Estrogen plays a key role in bone remodeling and growth and maintaining a balance between osteoblastic and osteoclastic activity in females [46]. Moreover, thyroid hormones play a pivotal role in linear development and maturation of the skeleton and in achieving peak bone mass [47]. Glyphosate can impact bone metabolism by interfering with key endocrine pathways that have downstream effects on bone metabolism. Many in vivo and in vitro studies have shown that glyphosate can disrupt hormonal pathways including hypothalamic-pituitary-gonadal axis in turn impacting other endocrine and reproductive endpoints [48]. Gasnier et al. (2009) used human liver cells to show that increasing dosing with glyphosate induced endocrine disruption including androgen receptors, estrogen receptors, cytotoxicity and DNA damage [49]. Some human evidence also exists for an endocrine disruption hypothesis. A recent publication by Geier et al. confirmed a significant inverse dose-dependent relationship between increasing concentrations of urinary glyphosate and decreasing serum total estrogen in middle-age adults (median age 40 years) [10]. Direct interference with calcium metabolism is also plausible. Recent research shows that once glyphosate enters the bloodstream, it quickly moves into bone marrow and bone, where it can bind to calcium and is stored [50]. Finally, a plausible mechanism could be prolonged distribution of glyphosate into the bone marrow increases its interaction with hematopoietic stem cells (HSCs), and mesenchymal stem cells (MSCs). Bone marrow is not only a source of HSCs, which are responsible for producing all blood cells, including immune cells, but also for MSCs which produce osteoblasts and osteocytes, the bone-forming and maintenance cells. Findings from animal bioassays and epidemiological studies support an association between glyphosate exposure and an increased risk of hematologic malignancies derived from HSCs, through oxidative stress, disruption of DNA repair among other potential pathologies [50, 51]. A similar mechanism could be at play for MSCs that are precursors to bone cell lines [18, 44, 52].

It is well known that the high risk population for detecting the evidence of an exposure on bone health is postmenopausal women [53], so it is not surprising that we found the greatest evidence of adverse glyphosate associations in this subpopulation within NHANES. Human bone accretion continues from childhood to adulthood, and peak bone mass in the spine and hip is typically achieved in the mid-twenties in females [20]. However, starting from the third decade, females lose 50% of their trabecular bone and 35% of the cortical bone [50], whereas in males, the bone loss over the lifetime is approximately two-thirds of the amount in females [54]. Of the many factors that influence bone loss, such as genetics and environment, sex hormone deficiency is considered to be the most important [53]. In females after midlife, the normal bone turnover cycle is impaired by gradually decreasing levels of estrogen [54]. The bone loss in early osteoporosis is mainly a trabecular bone loss, attributed to estrogen deficiency. However, within 4–8 years, the second phase with loss of both trabecular and cortical bone (hip) begins, mainly due to reduced bone formation.

Bone loss in females is important from a public health perspective as it is linked with fractures [54], consistent with our findings from NHANES data. Age-related bone loss decreases BMD which is the single best predictor of future risk of fractures [55]. It is estimated that 40% women will experience one or more fractures after the age of 50 years. For females at age 50 years, the lifetime risk is 17.5% for hip fracture and 16% for vertebral fracture; in comparison, for males the lifetime risks are 6% and 5%, respectively [55]. Fractures have a profound socioeconomic impact on an aging population including reduced life expectancy, prolonged medical care, and loss of independence. If the associations noted in our study are determined to be causal, including the association with fracture, the public health implications of a commonly applied pesticide would have another cause for reevaluation. Further, if there really is an adverse effect of cumulative glyphosate exposure on BMD, our estimates, based on recent exposure only, are in theory more likely to be attenuated than exaggerated. To our knowledge, this is also the first report assessing the relationship between glyphosate exposure and BMD in children [7]. Creatinine-adjusted glyphosate levels were higher in male children than in male adults in our study and in others [4]. While there are some speculations as to why male children have higher glyphosate levels than male adults, there has been no reported direct effect on bone health in children.

It is important to note that urinary glyphosate concentrations may vary considerably within individuals over time. Such intra-individual variability may lead to exposure misclassification and attenuate the observed associations (bias toward the null). Although creatinine adjustment and NHANES laboratory procedures improve measurement reliability, a single spot urine sample does not fully capture long-term exposure. Previous studies have also reported substantial within-person variability in urinary glyphosate levels [56].

The strengths of this study include a representative sample of the U.S. population, the inclusion of both children and adults of multiple races and ethnicities, and the capability to tie imaging biomarkers of bone density to the downstream outcomes of osteoporosis and fractures. However, there are also reasons to be cautious. The observed associations are not necessarily causal, and temporality of the associations could not be established despite the consistency of findings and the high degree of parallel biological plausibility for experimental bone toxicity of glyphosate. Our study has both clear limitations as well as findings of uncertain meaning. The adverse association between glyphosate exposure and BMD, osteoporosis, and fractures, although statistically significant, is based on cross-sectional data and relies on a single sample of random urine sample. While the ion chromatography coupled with tandem mass spectrometry (IC- MS/MS) used in NHANES provides a higher specificity and sensitivity in detecting the urine glyphosate levels than other methods including liquid chromatography-MS/MS^22^, we also acknowledge the limitation that capturing the exposure marker using a random urine sample could lead to exposure misclassification and bias) [57, 58] The measurement of urine as an exposure marker potentially reflects only recent exposure and is at most a limited indication of cumulative exposure. While our analysis accounted for dilution-dependent sample variation by adjusting for creatinine levels, 24-hour urine samples or a first morning urine sample may provide a better measurement of environmental glyphosate exposure. Despite differences in where people live or season when herbicides are used, levels of glyphosate in urine stay consistent in biomonitoring, and toxicokinetic studies as summarized in a recent review by Benbrook et al. [50]. According to this review, once glyphosate enters the bloodstream, it quickly moves into bone marrow and bone, where it can bind to calcium and become stored [50]. Over time, it slowly leaks back into the bloodstream and is excreted in urine. This slow release could explain why urine levels of glyphosate remain stable even with varying exposure. Although limited by cross sectional design, our study still offers some support to the recent evidence that the distribution of glyphosate in bone marrow, its interaction with calcium homeostasis and osteocytes in skeletal tissue could shed some light on the toxicokinetics of glyphosate in relation to bone health [58].

Other adjustments that could be considered were not performed, such as adjustments for diet (which could affect both bone mineral density and glyphosate levels and which is controversial as an adjustment in NHANES [31], as well as physical activity. which is difficult to address across age groups. The osteoporosis and fracture outcomes are based upon self-report in a very well-done survey (although it is hard to predict that any recall or reporting bias should relate differentially to measured urine glyphosate). Thus, the associations we have detected, however consistent and plausibly causal, should be confirmed by other means in further studies.

The high prevalence, severe complications, and high cost of osteoporosis and osteoporosis-related fractures mean that any contributing cause is of substantial public health importance [59]. Determining if these associations are causal is a priority. Looking ahead, emphasis should be placed on in vitro studies with human mesenchymal stem cells, bone-forming osteoblasts and bone resorbing osteoclasts to explore association with glyphosate further. In addition, longitudinal human population studies with robust estimates of cumulative exposure are needed, with an emphasis on enrollment of a subpopulation with predicted high exposure. In such studies, multiple measures of exposure are likely to be needed to develop a worthwhile model, and a priori kidney health should be carefully considered by several means. To our knowledge, no studies on repeated urine or serum measurements of glyphosate in relationship to human bone health exist, emphasizing the need to expand the research on possible impacts of chronic exposure.

Residual confounding remains possible, particularly from factors such as diet and physical activity that were not included in the models due to concerns about measurement error and overadjustment.

Self-reported osteoporosis and fracture outcomes may be affected by recall bias, which can lead to misclassification. In most cases, such misclassification is expected to be non-differential, likely biasing the associations toward the null. However, among postmenopausal women, who are more likely to be screened and better informed about their bone health, the potential for differential misclassification cannot be excluded, although it is not clear that such misclassification should be related to urine glyphosate. This subgroup-specific limitation should be considered when interpreting the observed associations.

Because of the cross-sectional design of NHANES, temporality cannot be asserted, and it is not possible to establish whether glyphosate exposure precedes the observed bone-related outcomes. Therefore, the associations reported here cannot be interpreted as causal and should be confirmed in future longitudinal or experimental studies with repeated measures of exposure.

## Policy implications and future strategies

If future longitudinal or experimental studies confirm a causal relationship between glyphosate exposure and adverse bone outcomes, several public health strategies could be considered. Regulatory efforts could target controlling the source of exposure by reducing the Acceptable Daily Intake (ADI) and Maximum Residue Limits (MRLs) for glyphosate, alongside implementing targeted use restrictions including ban on pre-harvest use as desiccant. Monitoring strategies may focus on occupational biomonitoring with urinary checks for high-risk workers at strategic intervals and integrating glyphosate exposure and bone health markers into population-level surveillance programs such as NHANES. Finally, intervention strategies in non-worker populations could involve consideration of modified and more intense screening for those who live in proximity to glyphosate applications, especially for postmenopausal women.

## Conclusion

This study shows an adverse association between glyphosate exposure and BMD, osteoporosis, and fractures, primarily in postmenopausal adult females. The reasonable consistency of findings and the parallel biological plausibility from experimental literature support a need for serious consideration of causation. Causation cannot be asserted, however, from this study due to its cross-sectional design and the use of a single acquisition of a biological marker of exposure that measures only very recent exposure. Future longitudinal studies should consider repeated measures due to the short half-life of glyphosate. The public health/socioeconomic costs of a causal association for this topic would be important, suggesting a need for future studies on bone health and glyphosate exposure in other populations.

## Data Availability

Data analyzed will be available from the corresponding author (Naila Khalil) on reasonable request.
